# Ridge, Lasso and Bayesian additive-dominance genomic models

**DOI:** 10.1186/s12863-015-0264-2

**Published:** 2015-08-25

**Authors:** Camila Ferreira Azevedo, Marcos Deon Vilela de Resende, Fabyano Fonseca e Silva, José Marcelo Soriano Viana, Magno Sávio Ferreira Valente, Márcio Fernando Ribeiro Resende, Patricio Muñoz

**Affiliations:** Department of Statistics, Universidade Federal de Viçosa, Viçosa, Minas Gerais Brazil; Embrapa Forestry, Colombo, Paraná Brazil; Department of Animal Science, Universidade Federal de Viçosa, Viçosa, Minas Gerais Brazil; Department of General Biology, Universidade Federal de Viçosa, Viçosa, Minas Gerais Brazil; RAPiD Genomics, Florida Innovation Hub, Gainesville, Florida USA; Agronomy Department, University of Florida, Gainesville, Florida USA

**Keywords:** Dominance genomic models, Bayesian methods, Lasso methods, Selection accuracy

## Abstract

**Background:**

A complete approach for genome-wide selection (GWS) involves reliable statistical genetics models and methods. Reports on this topic are common for additive genetic models but not for additive-dominance models. The objective of this paper was (i) to compare the performance of 10 additive-dominance predictive models (including current models and proposed modifications), fitted using Bayesian, Lasso and Ridge regression approaches; and (ii) to decompose genomic heritability and accuracy in terms of three quantitative genetic information sources, namely, linkage disequilibrium (LD), co-segregation (CS) and pedigree relationships or family structure (PR). The simulation study considered two broad sense heritability levels (0.30 and 0.50, associated with narrow sense heritabilities of 0.20 and 0.35, respectively) and two genetic architectures for traits (the first consisting of small gene effects and the second consisting of a mixed inheritance model with five major genes).

**Results:**

G-REML/G-BLUP and a modified Bayesian/Lasso (called BayesA*B* or t-BLASSO) method performed best in the prediction of genomic breeding as well as the total genotypic values of individuals in all four scenarios (two heritabilities x two genetic architectures). The BayesA*B*-type method showed a better ability to recover the dominance variance/additive variance ratio. Decomposition of genomic heritability and accuracy revealed the following descending importance order of information: LD, CS and PR not captured by markers, the last two being very close.

**Conclusions:**

Amongst the 10 models/methods evaluated, the G-BLUP, BAYESA*B* (−2,8) and BAYESA*B* (4,6) methods presented the best results and were found to be adequate for accurately predicting genomic breeding and total genotypic values as well as for estimating additive and dominance in additive-dominance genomic models.

## Background

The goal of genome-wide selection (GWS) is early phenotype prediction; it relies on simultaneously predicting the effects (on phenotype) of a large number of molecular markers. Thus, it represents a new paradigm in quantitative genetics [1, 2] and plant and animal breeding [3–7].

The simultaneous prediction of marker effects is now common in genome-wide association studies (GWAS) [8–10] also. As a result, GWS methods are also being used in human genetics, gene discovery and association genetics.

Recent methodologies for GWS and GWAS have been evaluated with simulation studies [11, 12]. Simulation and practical results with additive models in GWS with several organisms are common [13–17]. However, additive-dominance models are much less common [17–20].

Hill et al. [21], Bennewitz and Meuwissen [22] and Wellmann and Bennewitz [23] discussed the relevance of dominance models for Quantitative Genomics and Genetics. Wellmann and Bennewitz [23] presented theoretical genetic models for Bayesian genomic selection with dominance and concluded that dominance enhances the analysis and has several advantages. Wang and Da [24] established the correct definitions of genomic relationships and inbreeding, which came to unify the prediction models for additive-dominance genomic selection. Da et al. [25] and Wang et al. [26] presented a software for additive-dominance models in the framework of the G-BLUP method.

Dominance estimation is essential, especially for vegetative propagated species [20] and crossed populations, where including both additive effects and dominance in the mating allocation is an effective way of increasing genetic gain by capitalizing on heterosis [23, 27]. Additive-dominance models are able to capture both effects, allowing the effective selection of parents, crosses and clones. This process takes full advantage of genomic selection in perennials and asexually propagated crops, as well as in crossed animals.

Bayesian, Lasso and Ridge regression approaches have not yet been compared for additive-dominance models. [17–20] and [24] applied only the G-BLUP method, which is an equivalent model [10], to ridge regression (RR-BLUP). On the other hand, [23] applied only the Bayesian methods of [1] with modifications (a mixture of two t distributions, one of them having a small variance). Toro and Varona [27] evaluated the introduction of dominant effects into the model using Bayes A. Lasso methods seems to be unused in dominance models for variance components in genomic selection. The partitioning of accuracy and heritability due to the three forms of quantitative genetics information, including linkage disequilibrium (LD), co-segregation (CS) and pedigree relationships (PR), is an important subject and has only been explored by [28].

Given the scarcity of papers on dominance genomic models in the literature and for the purpose of increasing knowledge and enriching discussion of such an essential topic in this field, the objective of this paper is two-fold: (i) to evaluate 10 estimation methods (including the Bayesian, Lasso and Ridge regression approaches) for fitting additive-dominance genomic models for GWS; and (ii) to decompose genomic heritability and accuracy in terms of the three quantitative genetic information compounds LD, CS and PR.

## Methods

### Simulated datasets

Two random mating populations in linkage equilibrium were crossed generating a population (of size 5,000, coming from 100 families) with LD, which was subjected to five generations of random mating without mutation, selection or migration. The resultant population is an advanced generation composite, which presents Hardy-Weinberg equilibrium and LD. According to [29], the LD value in a composite population is ${\Delta}_{\mathrm{a}\mathrm{b}}\kern0.5em =\kern0.5em \left(\frac{1\kern0.5em -\kern0.5em 2{\uptheta}_{\mathrm{a}\mathrm{b}}}{4}\right)\;\left(\;{\mathrm{p}}_{\mathrm{a}}^1\kern0.5em -\kern0.5em {\mathrm{p}}_{\mathrm{a}}^2\right)\;\left(\;{\mathrm{p}}_{\mathrm{b}}^1\kern0.5em -\kern0.5em {\mathrm{p}}_{\mathrm{b}}^2\right)$, where a and b are two SNPs, two QTLs, or one SNP and one QTL, θ is the frequency of recombinant gametes, and p^1^ and p^2^ are the allele frequencies in the parental populations (1 and 2). Notice also that the LD value depends on the allele frequencies in the parental populations. Thus, regardless of the distance between the SNPs and/or QTLs, if the allele frequencies are equal in the parental population, Δ = 0. The LD is maximized (|Δ| = 0.25) when θ = 0 and |p^1^ - p^2^| = 1. In this case, the LD value is positive with coupling and negative with repulsion [30].

From the advanced generation of the composite, 1,000 individuals were generated with diploid genomes having a length of 200 centimorgans (cM) (L = 2 Morgans) and assuming ten equally sized chromosomes, each one with two haplotypes. We simulated a marker density by assigning 2,000 equidistant SNP markers that were separated by 0.1 cM across the ten chromosomes. One hundred of the 2,000 markers were actually genes (QTL). A total of 1,000 individuals that came from the same generation and from 20 full-sib families (each one with 50 individuals) were genotyped and phenotyped. This simulation provides a typical small effective population size (Ne = 39.22) and a large LD in the breeding populations. Ne of approximately 40 and the use of 50 individuals per family are typical values in elite breeding populations of plant species.

The QTLs were distributed in the regions covered by the SNPs. For each trait, we informed the degree of dominance (*d*/*a*) and the direction of dominance (positive and/or negative). The obtained genotypic values for homozygotes were within the limits of *Gmax = 100(m + a)* and *Gmin = 100(m - a)*, which are the maximum and minimum values, respectively.

Goddard et al. [31] presented the realized proportion (*r*_*mq*_^2^) of genetic variation explained by the markers as ${r}_{mq}^2=\frac{n}{n+{n}_{QTL}}$, where *n*_*QTL*_ is the number of QTL. With *n* = 2,000 markers and *n*_*QTL*_ = 100, we have *r*_*mq*_^2^ = 0.95. An alternative [14] takes *n*_*QTL*_ = 2*NeL* = 2 39.22 2 = 156.88, producing *r*_*mq*_^2^ = 0.93. Another approach [32] provides *r*_*mq*_^2^ as ${r}_{mq}^2=\frac{1}{1+4NeS}=\frac{1}{1+4\kern0.5em 39.22\kern0.5em 0.001}=0.86.$ L is the total length of the genome, and S is the spacing between markers (both in Morgans). These values reveal that the genome was sufficiently saturated by markers.

Traits with two genetic architectures were simulated, one following the infinitesimal model and the other with five major effects genes accounting for 50 % of the genetic variability. For the former, to each of 100 QTL one additive effect of small magnitude on the phenotype was assigned (under the Normal Distribution setting). For the latter, small additive effects were assigned to the remaining 95 loci. The effects were normally distributed with zero mean and variance, allowing the desired heritability level. The phenotypic value was obtained by adding to the genotypic value a random deviate from a normal distribution N (0, *σ*_*e*_^2^), where the variance *σ*_*e*_^2^ was defined according to two levels of broad-sense heritability, 0.30 and 0.50, associated with narrow-sense heritabilities of approximately 0.20 and 0.35, respectively. Heritability levels were chosen to represent one trait with low heritability and another with moderate heritability, which addressed the cases where genomic selection is expected to be superior to phenotypic selection. The magnitudes of the narrow-sense and broad-sense heritabilities are associated with an average degree of dominance level (d/a) of approximately 1 (complete dominance) in a population with intermediate allele frequencies. Simulations assumed independence of additive and dominance effects, with dominance effects having the same distribution as the additive effects (both were normally distributed with zero mean). In the simulation, it was also observed that marker alleles had MAF (minor allele frequency) greater than 5 %.

#### Scenarios

For the populations of full-sib families, four scenarios were studied: two broad-sense heritability levels (approximately 0.30 and 0.50) × two genetic architectures. The scenarios were analyzed using 10 statistical methods (Table 1).

**Table 1:** Softwares

Method	Full name of the method	Class of methods	DF1	DF2	Software
BRR (−2,-2)	Bayesian Ridge Regression	Bayesian	−2	−2	GS3
IBLASSO (4,-2)	Improved Bayesian Lasso	Bayesian Lasso	4	−2	GS3
IBLASSO (4,2)	Improved Bayesian Lasso	Bayesian Lasso	4	2	GS3
BAYESA*B* (−2,6)	IBLASSO with t distribution	Bayesian Lasso	−2	6	GS3
BAYESA*B* (4,6)	IBLASSO with t distribution	Bayesian Lasso	4	6	GS3
BAYESA*B* (−2,8)	IBLASSO with t distribution	Bayesian Lasso	−2	8	GS3
RR-HET (-2,–2)	RR-BLUP with heterogeneous variance	Ridge Regression	-2	−2	GS3
BLASSO (4,2)	Bayesian Lasso	Bayesian Lasso	4	2	BLR-R
G-BLUP	Genomic BLUP	Random Regression	-	-	GVC
Pedigree-BLUP	Pedigree-BLUP	Random Regression	-	-	Pedigreem-R

Description of the fitted models and softwares used

DF1: Degrees of Freedom of the chi-square prior distribution for the residual variance;

DF2: Degrees of Freedom of the chi-square prior distribution for genetic variance or shrinkage parameter

### Additive-dominance model for the REML/G-BLUP method

A mixed linear model for individual additive breeding values (*u*_*a*_) and dominance deviations (*u*_*d*_) is as follows: *y* = *Xb* + *Zu*_*a*_ + *Zu*_*d*_ + *e*, with the variance structure given by ${u}_a\sim N\left(0,{G}_a{\sigma}_{u_a}^2\right)$; ${u}_d\sim N\left(0,{G}_d{\sigma}_{u_d}^2\right)$; *e* ~ *N*(0, I*σ*_*e*_^2^). An equivalent model [33] at the marker level is given by *y* = *Xb* + *ZWm*_*a*_ + *ZSm*_*d*_ + *e*, where:

$$ \begin{array}{l}{u}_a=W{m}_a;\kern0.5em \\ {}Var\left(W{m}_a\right)=WI{\sigma}_{m_a}^2W\hbox{'}=WW\hbox{'}{\sigma}_{m_a}^2;\\ {}{u}_d=S{m}_d;\\ {}\kern0.5em Var\left(S{m}_d\right)=SI{\sigma}_{m_d}^2S\hbox{'}=SS\hbox{'}{\sigma}_{m_d}^2.\end{array} $$

W and S are the incidence matrices for the vectors of additive (*m*_*a*_) and dominance (*m*_*d*_) marker genetic effects. The variance components associated to these effects are ${\sigma}_{m_a}^2$ and ${\sigma}_{m_d}^2$, respectively. *G*_*a*_ and *G*_*d*_ are the genomic relationship matrices for the additive and dominance effects. The quantity *m*_*a*_ in one locus is the allele substitution effect and is given by *m*_*ai*_ = *α*_*i*_ = *a*_*i*_ + (*q*_*i*_ − *p*_*i*_)*d*_*i*_, where p_i_ and q_i_ are allelic frequencies and *a*_*i*_ and *d*_*i*_ are the genotypic values for one homozygote and heterozygote, respectively, at locus i. In turn, the quantity *m*_*d*_ can be directly defined as *m*_*di*_ = *d*_*i*_.

The matrices *W* and S, which will be defined later, are based on the values 0, 1 and 2 for the number of one of the alleles at the *i* marker locus (putative QTL) in a diploid individual. Several parameterizations are available, and the one that matches well with classical quantitative genetics theory [34] is as follows [5, 24, 25, 35].

The correct parameterization of W and S is as follows, according to the marker genotypes at a locus m.

1 $$ W=\left\{\begin{array}{l}\mathrm{If}\kern0.24em \mathrm{M}\mathrm{M},\ \mathrm{then}\kern0.48em 2-2p\kern0.6em \to 2q\\ {}\mathrm{If}\;\mathrm{M}\mathrm{m},\ \mathrm{then}\kern0.5em 1\kern0.36em -2p\kern0.48em \to q-p\\ {}\mathrm{If}\kern0.24em \mathrm{m}\mathrm{m},\ \mathrm{then}\kern0.36em 0-2p\kern0.6em \to -2p\end{array}\right. $$

2 $$ S=\left\{\begin{array}{l}\mathrm{If}\kern0.24em \mathrm{M}\mathrm{M},\ \mathrm{then}\kern0.24em 0\kern0.48em \to -2{q}^2\\ {}\mathrm{If}\kern0.24em \mathrm{M}\mathrm{m},\ \mathrm{then}\kern0.48em 1\to 2pq\\ {}\mathrm{If}\kern0.24em \mathrm{m}\mathrm{m},\ \mathrm{then}\kern0.6em 0\kern0.36em \to -2{p}^2\end{array}\right. $$

The covariance matrix for the additive effects is given by *G*_*a*_*σ*_*a*_^2^ = *Var*(*Wm*_*a*_) = *WW* ' *σ*_*ma*_^2^, which leads to ${G}_a=WW\hbox{'}/\left({\sigma}_{ma}^2/{\sigma}_a^2\right)=WW\hbox{'}/{\displaystyle \sum_{i=1}^n\left[2{\mathrm{p}}_i\left(1-{p}_i\right)\right]}$, as ${\sigma}_a^2={\displaystyle \sum_{i=1}^n\left[2{\mathrm{p}}_i\left(1-{p}_i\right)\right]}{\sigma}_{ma}^2$. The covariance matrix for the dominance effects is given by *G*_*d*_*σ*_*d*_^2^ = *Var*(*Sm*_*d*_) = *SS* ' *σ*_*md*_^2^. Thus, ${G}_d=SS\hbox{'}/\left({\sigma}_{md}^2/{\sigma}_d^2\right)=SS\hbox{'}/{\displaystyle \sum_{i=1}^n{\left[2{\mathrm{p}}_i\left(1-{p}_i\right)\right]}^2}$, as ${\sigma}_d^2={\displaystyle \sum_{i=1}^n{\left[2{\mathrm{p}}_i\left(1-{p}_i\right)\right]}^2}{\sigma}_{md}^2$.

The additive-dominance G-BLUP method was fitted using GVC-BLUP software [26] via REML through mixed model equations.

### Bayesian Ridge Regression (BRR) method

A Bayesian additive-dominance G-BLUP or Bayesian Ridge Regression (BRR) method was fitted using GS3 software [36] via MCMC-REML/BLUP assigning flat (i.e., with degrees of freedom equal to −2, which turns the inverted chi-square into a uniform distribution) prior distributions for variance components. (The *a priori* flat is a the noninformative one).

### BayesA and BayesB methods

The BayesA and BayesB methods, described by [1], are advantageous because they can potentially provide information on the genetic architecture of the quantitative trait.

In these methods, specific variances are allowed at each locus. Additionally, BayesB performs variable selection because the majority of the markers are not in LD with the genes. Thus, a set of markers associated with a trait must be identified. The BayesB method subjectively determines π, the proportion of markers having effects. Using the indicator variable I, in the BayesA and BayesB models, the additive genetic effect of an individual j is defined as ${a}_j={\displaystyle \sum_{i=1}^n{m}_{ai}{w}_{ij}{I}_{ai}}$, where *I*_*ai*_ = (0, 1). The distribution of *I*_*a*_ = (*I*_*a*1_ … *I*_*an*_) is binomial with a probability π, which is 1 for BayesA and is subjectively determined for BayesB. The quantities of *w*_*ij*_ are elements of the marker genotype matrix W. Dominance effects are coded in a similar way: ${d}_j={\displaystyle \sum_{i=1}^n{m}_{di}{s}_{ij}{I}_{di}}$.

These Bayesian methods assume that the conditional distribution of each marker effect (given its variance) follows a normal distribution, i.e., *m*_*ai*_|*σ*_*mai*_^2^ ~ *N*(0, *σ*_*mai*_^2^). The variances of the marker effects are assumed to be a scaled inverse chi-square distribution with v degrees of freedom and scale parameter ${S}_{m_a}^2$, i.e., *σ*_*mai*_^2^ ~ *χ*^− 2^(*ν*_*ma*_, *S*_*ma*_^2^). This assumption implies that a larger number of markers has small effects and a small number of markers has large effects, which leads to a univariate t-distribution of the marker effects with mean zero [37]. Gianola et al. [2] proved that fitting a variance by locus in this way is equivalent to postulating a *t* distribution for all loci. Thus, the identification of relevant marker effects is more likely in the t-BayesA model than in the normal-RR-BLUP model.

For the Bayes methods, the marginal prior distribution for additive marker effects is ${m}_{ai}\Big|{\nu}_{m_a},{S}_{m_a}^2\sim t\left(0,{\nu}_{m_a},{S}_{m_a}^2\right)$. The combination of normal (for marker effects) and inverse chi-square distributions (for variances) leads to a t distribution for *m*_*ai*_, and thus a longer tail than that for normal distribution. In this paper, the values 6 and 8 were assigned for v to provide sufficiently thick tails associated to t distributions [38], and ${S}_{m_a}^2$ was calculated from the additive variance according to the method of [39].

For dominance effects at the intra-population level, the distributions are similar to what was described for additive effects. Thus, *m*_*di*_|*σ*_*mdi*_^2^ ~*N*(0, *σ*_*mdi*_^2^) for the marker dominance effects; *σ*_*mdi*_^2^ ~*χ*^− 2^(*ν*_*md*_, *S*_*md*_^2^) for the marker dominance variance, with the marginal of the prior distribution for marker dominance effects given by ${m}_{di}\Big|{\nu}_{m_d},{S}_{m_d}^2\sim t\left(0,{\nu}_{m_d},{S}_{m_d}^2\right)$.

Additive and dominance variances are given by ${\sigma}_a^2={\displaystyle \sum_{i=1}^n2{\mathrm{p}}_i\left(1-{p}_i\right)}{m}_{ai}^2$ and ${\sigma}_d^2={\displaystyle \sum_{i=1}^n{\left[2{\mathrm{p}}_i\left(1-{p}_i\right)\right]}^2}{m}_{di}^2$, respectively, according to the parameterizations in W and S. The full conditional distributions for the parameters of the BayesA and BayesB models were presented in detail by [18].

### BayesA*B* or IBLASSO_t_ method

According to [40], a strong influence of prior parameters on predictive ability was observed in the BayesA and BayesB models. Variation in the scale parameters *S*_*ma*_^2^ and *S*_*md*_^2^ in these methods had a strong impact on prediction. An overlarge scale (*S*_*ma*_^2^ or *S*_*md*_^2^) for the prior distribution of variance led to overfitting of the data, while a scale parameter that was too small led to underfitting due to excessive shrinkage of the effects. In both cases, the predictive ability is considerably reduced. Consequently, to obtain good predictive abilities, an appropriate choice of hyperparameters is necessary to prevent both over- and underfitting.

The differences between the explicit regression GWS methods are mainly due to the type and extent of the shrinkage imposed by the method, the ability to learn from the data, and the influence of prior distributions. In the case of N < <<n (n is the number of markers and N is the number of individual observations), learning from the data is difficult to verify because the data (likelihood) do not dominate the posterior distribution. Thus given the same sampling model postulated by the methods, likelihood shrinkage properties are not very different. Thus, any differences in posterior inferences between these methods must be because priors are influential and very different [38]. Based on this analysis, it can be asserted that different methods can be fitted with the same machinery only by somehow drastically altering the prior distribution.

The Bayesian Lasso method provides better learning from the data than BayesA and BayesB [2, 38]. The difference between the Bayesian LASSO and the Bayesian approaches (BayesA and BayesB) developed by [1] is derived from the different specifications of the prior variance of the marker-specific regression coefficient as well as the type and extent of shrinkage effected.

For this reason, we chose to implement BayesA using the BLASSO framework by specifying the *prior* distribution through appropriate degrees of freedom (6 and 8) for the scaled inverse chi-square distribution associated with marker genetic variance (and then with the penalization parameter *λ*). This produces a t-like distribution, which is an intermediate between the normal (of the RR-BLUP) and double exponential (of the Lasso) distributions and provides desirable shrinkage estimates for the QTL effects, as does BayesA.

By fitting in this way (via BLASSO), BayesA has better learning properties. This improved BayesA can be called BayesA* and can turn out to be Bayes B* if the BLASSO machinery effectively leads a large number of markers to zero effects. In this case, the method will be called Bayes A*-B* (or t-Bayesian Lasso) because it conjugates the priors of BayesA and the type and extent of shrinkage (covariable selection) of the Blasso method. In their fast BayesB method, [41] changed the prior distribution of marker effects from a Student-t distribution to a double exponential of Laplace, which improved the model and perhaps made it closer to the BLASSO method. Kärkkäinen and Sillanpää [42] discussed the interchange of Student-t and Laplace (DE) as prior distributions of marker effects. Another possible name for Bayes A*-B* is t-BLASSO, meaning Bayesian Lasso [43] with a t distribution as the prior for marker effects.

Bayes A*-B* methods were fitted using GS3 software [36] via MCMC assigning with 6 and 8° of freedom for the inverted chi-square distribution for genetic variance (and then with the penalization parameter *λ*), which converts the prior for marker effects into a t distribution. This approach is expected to produce results similar to the Bayes methods of [1] but with the learning ability of the BLASSO method. Additionally, the BLASSO is asymptotically free of prior information and more consistent than BayesB and does not require tuning.

### BLASSO and IBLASSO methods

In the Bayesian Lasso [44], the prior assigned to marker effects is a Laplace (double exponential, DE) distribution. All marker effects are assumed to be independently and identically distributed as a DE. This prior assigns the same variance or prior uncertainty to all marker effects, but it possesses thicker tails than the normal or Gaussian prior. Comparative discussions of the DE prior are in [45] and [46].

With two variance components (*σ*_*e*_^2^ and *σ*_*ma*_^2^), the model is called an improved Bayesian Lasso (IBLASSO) [43]. The practical implementation of this model via Gibbs sampling, including the full *posterior* conditional distributions, was described by [43]. For dominance effects, similar distributions hold as described for additive effects.

Concerning the IBLASSO of [43], [38] criticizes the choice of a uniform flat prior on the regularization parameter *λ*. Because of this criticism, our paper used two alternative priors: a similar flat prior and also a prior with 4° of freedom on the parameter *λ*, as in the case of the BLASSO. Computations were performed in the GS3 Software.

### Ridge Regression with heterogeneity of variances (RR-HET)

An additive-dominance Ridge Regression (RR-BLUP) method can also be implemented that considers the heterogeneity of variances between markers, called RR-HET. In our paper, the matrices with specific variances for each marker, *D*_*a*_ = *diag*(*τ*_1*a*_^2^, *τ*_2*a*_^2^, …, *τ*_*na*_^2^) and *D*_*d*_ = *diag*(*τ*_1*d*_^2^, *τ*_2*d*_^2^, …, *τ*_*nd*_^2^), were obtained by the BLASSO method (4, −2) using GS3 software.

### Fitting models

Each type of population was simulated 10 times under the same parameter settings, which preserved the same features and provided samples that were effectively of the same conceptual population. Nine replicates were used as training populations, and one replicate was used as a validation population. The estimations based on each of the nine replicates were validated by obtaining estimates of the parameters accuracy and bias. Validation and reference individuals belonged to the same population but to different families.

In each replicate, marker effects were estimated and used to estimate the genetic values of individuals in the tenth population. These estimated genetic values were correlated with the parametric genetic values of individuals of the tenth population, providing the accuracy values. The results from the nine analyses were averaged across replicates to obtain final accuracies and heritabilities for each scenario.

Methods for computing parametric accuracies under the additive-dominance models were derived following the method of [6]. The following formulas were obtained:

**Additive accuracy**: $$ {r}_{a\widehat{a}}=\sqrt{\frac{r_{mq}^2\left(N{r}_{mq}^2{h}_a^2/{n}_{QTL}\right)}{1+N{r}_{mq}^2{h}_g^2/{n}_{QTL}}} $$

**Dominance accuracy:**$$ {r}_{d\widehat{d}}=\sqrt{\frac{r_{mq}^2\left(N{r}_{mq}^2{h}_d^2/{n}_{QTL}\right)}{1+N{r}_{mq}^2{h}_g^2/{n}_{QTL}}} $$

**Genotypic accuracy:**$$ {r}_{g\widehat{g}}=\sqrt{r_{a\widehat{a}}^2+{r}_{d\widehat{d}}^2} $$,

where n_QTL_ is the number of QTL, N is the number of individuals in the estimation dataset, and *h*_*a*_^2^, *h*_*d*_^2^ and *h*_*g*_^2^ are additive, dominance and total heritability, respectively.

**Fig. 1:**
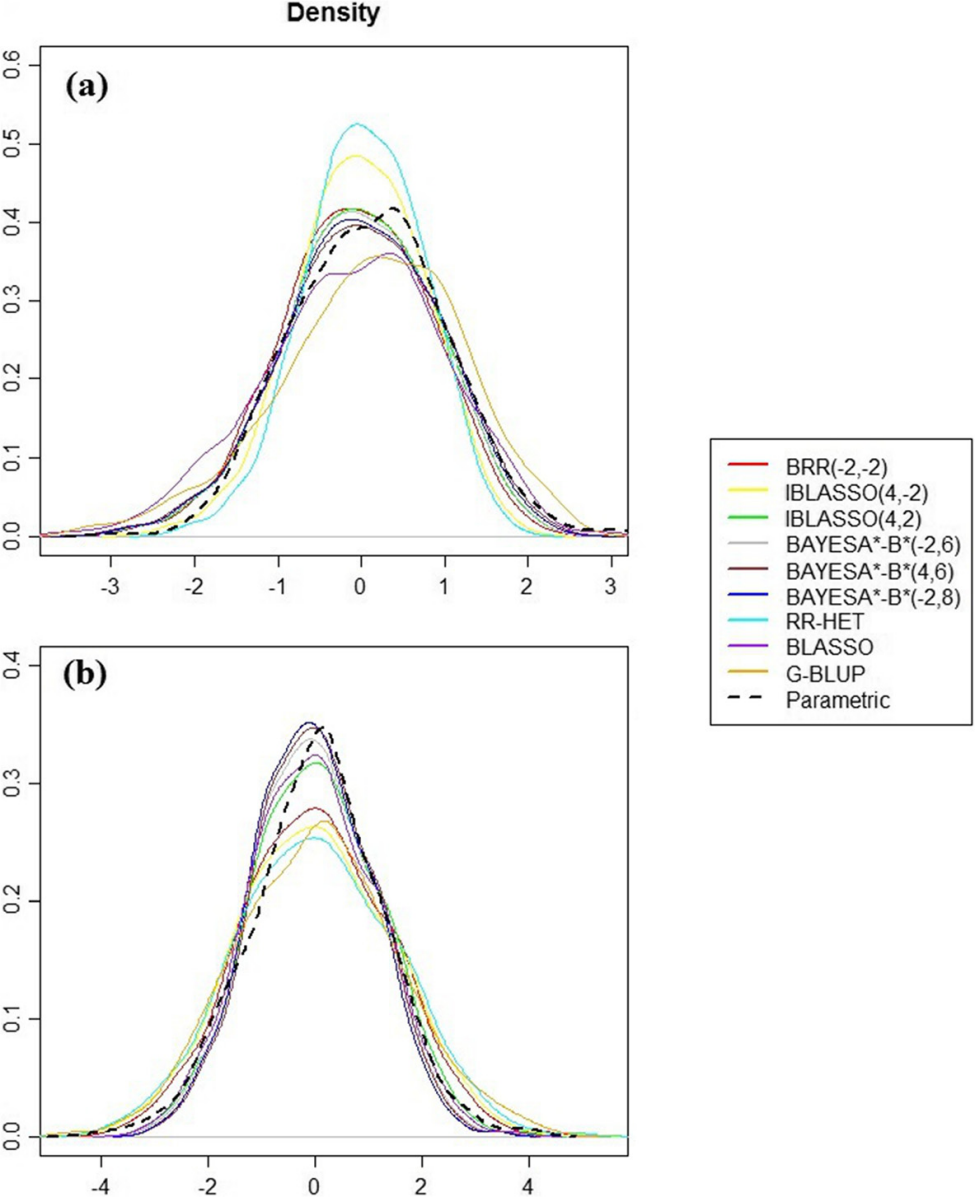
Posterior distributions. Parametric and predicted additive (**a**) and dominance (**b**) individual values (h2 = 0.30; small gene effects model)

For Bayesian methods, we used 120,000 iterations for the MCMC algorithms of the different models, with the first 20,000 iterations discarded as burn in. After every set of 10 iterations (thin) were performed, a sample was retained to calculate *a posteriori* statistics. Hence, 10,000 MCMC samples were used to construct the posterior densities. The convergence of the Markov chains was checked with a [47] diagnostic and also by visualizing the trace plot and running repeated progressive analyses until convergence was met. Posterior distributions were plotted (Fig. 1) to view the Bayesian learning of the methods. A summary of the fitted models is presented in Table 1.

### Decomposing the quantitative genetic information

The three types of quantitative-genetic information can be defined as in [28]:

*Linkage disequilibrium*: refers to founder alleles from different loci in the same gamete, and the loci are in LD (not sampled independently, i.e., in population level disequilibrium) and describe genetic relationships between founders.

*Co-segregation*: refers to non-founder alleles (not in LD and not identical by descent from the base population) from different loci in the same gamete, and the loci are linked (not transmitted independently, i.e., in population level equilibrium but in within-family level disequilibrium).

*Genetic relationships*: statistical dependency between alleles from the same locus in different gametes. This kind of information is of three types: When associated with markers, it refers to parentage only on the marker loci and does not involving a linkage between markers and QTL; when associated with the pedigree of individuals in a model with both markers and pedigree, it refers to residual polygenic effects; when associated with the pedigree of individuals only, it refers to total polygenic effects.

G-BLUP makes use of the following: (i) co-segregation of QTL and markers due to linkage; (ii) pedigree genetic relationships between markers not linked to QTL; and (iii) LD between markers and genes to capture relationships at QTL [28]. The genomic relationship matrix is called the realized relationship, as it describes IBD at SNP, assuming an ancient founder population. However, only genetic relationships at QTL matter.

The genomic relationship matrix includes LD, co-segregation and pedigree genetic relationships between markers not linked to QTL (for example, in structured populations). Habier et al. [28] derived formulas for proving that all three sources of information are used by G-BLUP.

The data sets analyzed were as follows: overall (raw or without any correction of the phenotypes); within-family deviations across families (with correction of the phenotypes for family effects and analyzing families altogether); and within each family with posterior averaging (with correction of the phenotypes for family effects and analyzing one family at a time). The accuracy of genomic selection in the analysis using the within-each-family with posterior averaging dataset is due to LD and co-segregation. In the analysis using the dataset from within-family deviations across families, the accuracy is due only to LD, while the accuracy of the analysis with the overall dataset is due to family IBD relationships, LD and co-segregation.

## Results

### Comparison of methods

In the evaluation of the methods, the following quantities were subjected to comparisons: heritability and dominance/additive variation ratio (the best are the closest to being parametric); accuracies (the highest values are the best); and bias (values closest to 1 are the best).

The results concerning the trait controlled by small gene effects with a heritability of 0.30 are presented in Table 2. It can be observed that, of the 10 methods, the BAYESA*B* (−2,8) method (or tBLASSO) had the seven best (b) criteria among the 7 classification criteria, followed by BAYESA*B* (4,6), which had six best criteria. The G-BLUP method fitted through GVC-REML was intermediate and seemed to overestimate the dominance/additive variation ratio slightly. Other intermediate methods were BRR (−2,-2) and BAYESA*B* (−2,6).

**Table 2:** Scenario 1: Results for the trait controlled by small gene effects with heritability 0.30

Method	h2a	h2d	cor_a	byg_a	cor_d	byg_d	Vd/Va	Number of criteria scored as best
Parametric	0.21 ± 0.01	0.10 ± 0.01	0.68	-	0.48	-	0.48	-
BRR (−2,-2)	0.15^b^ ± 0.05	0.12^b^ ± 0.05	0.63^b^ ± 0.03	1.40 ± 0.33	0.31^b^ ± 0.07	0.57^b^ ± 0.23	0.77	5^b^
IBLASSO (4,-2)	0.12 ± 0.06	0.14 ± 0.05	0.62^b^ ± 0.03	2.41 ± 1.82	0.28 ± 0.06	0.46 ± 0.24	1.19	1
IBLASSO (4,2)	0.14 ± 0.06	0.10^b^ ± 0.06	0.63^b^ ± 0.03	1.86 ± 1.14	0.29^b^ ± 0.06	0.63^b^ ± 0.42	0.81	4
BAYESA*B* (−2,6)	0.15^b^ ± 0.06	0.10^b^ ± 0.05	0.63^b^ ± 0.03	1.51 ± 0.57	0.29^b^ ± 0.06	0.69^b^ ± 0.42	0.67	5^b^
BAYESA*B* (4,6)	0.15^b^ ± 0.06	0.10^b^ ± 0.05	0.63^b^ ± 0.03	1.49^b^ ± 0.56	0.29^b^ ± 0.06	0.71^b^ ± 0.43	0.65	6^b^
BAYESA*B* (−2,8)	0.15^b^ ± 0.05	0.09^b^ ± 0.05	0.63^b^ ± 0.03	1.44^b^ ± 0.47	0.29^b^ ± 0.06	0.72^b^ ± 0.42	0.61^b^	7^b^
RR-HET (-2–2)	0.11 ± 0.06	0.14 ± 0.05	0.62^b^ ± 0.03	2.43 ± 1.74	0.28 ± 0.05	0.44 ± 0.23	1.24	1
BLASSO (4,2)	0.17^b^ ± 0.09	0.13 ± 0.02	0.63^b^ ± 0.03	1.44 ± 0.65	0.29^b^ ± 0.05	3.20 ± 5.34	0.74	3
G-BLUP	0.15^b^ ± 0.05	0.13 ± 0.06	0.63^b^ ± 0.03	1.25^b^ ± 0.35	0.31^b^ ± 0.04	0.70^b^ ± 0.30	0.83	5^b^
Pedigree	0.16^b^ ± 0.03	0.07 ± 0.01	0.53 ± 0.03	0.96^b^ ± 0.19	0.05 ± 0.02	0.20 ± 0.11	-	2

^b^best = highest + − 0.02 for h2a, h2d, cor a, cor d and Vd/Va; 0.5 to 1.5 for bya and byd; highest minus 2 for best criteria in the last column

The additive accuracies for alternative methods were 0.68, 0.63 and 0.53 for parametric GWS, GWS by the best methods and pedigree, respectively. The expected additive accuracy estimate of the parametric GWS obtained using a deterministic formula is 0.68 in this case. BayesA*B* methods and the G-BLUP method fitted using GVC-REML software were the best and gave accuracies of 0.63, which is close to the parametric case. These results reinforce the value of GWS, which performed better than the pedigree phenotypic selection (Table 2).

Figure 1 also corroborates the power of GWS in catching up to the parametric individual genetic values (in dark). The methods that fitted and best matched the parametric values were the BayesA*B*-type methods and the Bayesian Regression (in dark blue, brown, gray, red and green), as seen for additive effects in Fig. 1. For dominance effects, the best methods were the BayesA*B*-type methods (in dark blue, brown, gray). The Bayesian Regression (in red and green) did not follow these methods for the dominance effects.

In general, compared to the parametric values, the methods for additive-dominance models slightly underestimate the narrow sense heritability. The G-BLUP fitted via GVC software slightly overestimated the dominance heritability. The best methods were able to sufficiently capture the dominance heritability but were not completely able to capture the additive heritability, perhaps due to a limited number of markers and/or imperfect LD. Dominance heritability was overestimated by G-BLUP and BLASSO and perfectly estimated by BayesA*-B*.

Results concerning the trait controlled by a mixed (major and small gene effects) inheritance model with a heritability of 0.30 are presented in Table 3. It can be seen that the best methods were similar to the small gene size effects case (Table 2), except that the G-BLUP method fitted through GVC-REML software outperformed the three BayesA*B* methods. G-BLUP was better for estimating dominance effects, and the BayesA*B* methods were better for estimating the dominance/additive variation ratio. Such methods proved to be robust to the genetic architecture of the trait.

**Table 3:** Scenario 2: Results for the trait controlled by mixed (major and small gene effects) inheritance model with heritability 0.30

Method	h2a	h2d	cor_a	byg_a	cor_d	byg_d	Vd/Va	Number of criteria best
Parametric	0.20 ± 0.01	0.13 ± 0.01	0.65	-	0.53	-	0.64	-
BRR (−2,-2)	0.13^b^ ± 0.03	0.12^b^ ± 0.06	0.63^b^ ± 0.03	1.53^b^ ± 0.29	0.33 ± 0.04	0.65 ± 0.22	0.94	4^b^
IBLASSO (4,-2)	0.10 ± 0.04	0.14^b^ ± 0.05	0.64^b^ ± 0.03	3.49 ± 4.49	0.31 ± 0.04	0.55 ± 0.24	1.44	2
IBLASSO (4,2)	0.12^b^ ± 0.04	0.11^b^ ± 0.05	0.63^b^ ± 0.03	2.26 ± 2.22	0.32 ± 0.05	0.71^b^ ± 0.33	0.93	4^b^
BAYESA*B* (−2,6)	0.13^b^ ± 0.04	0.10 ± 0.04	0.63^b^ ± 0.03	1.53^b^ ± 0.53	0.33 ± 0.04	0.80^b^ ± 0.32	0.73	4^b^
BAYESA*B* (4,6)	0.13^b^ ± 0.04	0.10 ± 0.04	0.63^b^ ± 0.03	1.54^b^ ± 0.53	0.33 ± 0.04	0.79^b^ ± 0.32	0.74	4^b^
BAYESA*B* (−2,8)	0.14^b^ ± 0.04	0.09 ± 0.04	0.63^b^ ± 0.03	1.47^b^ ± 0.48	0.33 ± 0.04	0.83^b^ ± 0.33	0.68^b^	5^b^
RR-HET (-2–2)	0.10 ± 0.04	0.14^b^ ± 0.05	0.64^b^ ± 0.03	3.43 ± 4.38	0.31 ± 0.04	0.55 ± 0.24	1.43	2
BLASSO (4,2)	0.10 ± 0.03	0.16 ± 0.07	0.63^b^ ± 0.04	1.91 ± 0.82	0.32 ± 0.05	0.76^b^ ± 0.61	1.63	2
G-BLUP	0.14^b^ ± 0.03	0.13^b^ ± 0.03	0.64^b^ ± 0.04	1.26^b^ ± 0.21	0.38^b^ ± 0.04	0.84^b^ ± 0.20	0.92	6^b^
Pedigree	0.13^b^ ± 0.02	0.09 ± 0.01	0.46 ± 0.04	0.89^b^ ± 0.11	0.06 ± 0.03	0.22 ± 0.10	-	2

^b^best = highest + − 0.02 for h2a, h2d, cor a, cor d and Vd/Va; 0.5 to 1.5 for bya and byd; highest minus 2 for best criteria in the last column

Results concerning the trait controlled by small gene effects with a heritability of 0.50 are presented in Table 4. It can be seen from Table 4 that the best methods were the same as in Tables 2 and 3, i.e., the three BayesA*B* methods and the G-BLUP method fitted through GVC-REML software. The methods were good for estimating both additive and dominance effects as well as the dominance/additive variation ratio. As expected, accuracies for h^2^ = 0.5 were higher than for h^2^ = 0.3 (Table 2). The expected additive accuracy estimate of GWS obtained by a deterministic formula is 0.73 in this case. BayesA*B* methods and the G-BLUP method fitted through GVC-REML software were the best, with an accuracy of 0.70.

**Table 4:** Scenario 3: Results for the trait controlled by equal gene effects with heritability 0.50

Method	h2a	h2d	cor_a	byg_a	cor_d	byg_d	Vd/Va	Number of criteria best
Parametric	0.35 ± 0.01	0.17 ± 0.01	0.73	-	0.51	-	0.48	-
BRR (−2,-2)	0.25^b^ ± 0.04	0.20^b^ ± 0.03	0.69^b^ ± 0.03	1.42^b^ ± 0.23	0.36 ± 0.04	0.54^b^ ± 0.11	0.81	5^b^
IBLASSO (4,-2)	0.22 ± 0.06	0.22 ± 0.04	0.69^b^ ± 0.03	1.74 ± 0.82	0.35 ± 0.04	0.48 ± 0.11	1.01	1
IBLASSO (4,2)	0.24 ± 0.06	0.20^b^ ± 0.04	0.69^b^ ± 0.03	1.60 ± 0.71	0.36 ± 0.04	0.54^b^ ± 0.14	0.82	3
BAYESA*B* (−2,6)	0.25^b^ ± 0.06	0.18^b^ ± 0.04	0.70^b^ ± 0.03	1.53^b^ ± 0.66	0.36 ± 0.04	0.57^b^ ± 0.15	0.73^b^	6^b^
BAYESA*B* (4,6)	0.25^b^ ± 0.06	0.18^b^ ± 0.04	0.70^b^ ± 0.03	1.52^b^ ± 0.66	0.36 ± 0.04	0.58^b^ ± 0.15	0.72^b^	6^b^
BAYESA*B* (−2,8)	0.26^b^ ± 0.06	0.18^b^ ± 0.04	0.70^b^ ± 0.03	1.51^b^ ± 0.64	0.36 ± 0.04	0.59^b^ ± 0.15	0.69^b^	6^b^
RR-HET (-2–2)	0.22 ± 0.06	0.22 ± 0.04	0.69^b^ ± 0.03	1.76 ± 0.83	0.35 ± 0.04	0.48 ± 0.11	1.02	1
BLASSO (4,2)	0.18 ± 0.05	0.29 ± 0.03	0.69^b^ ± 0.03	1.69 ± 0.45	0.35 ± 0.03	0.46 ± 0.08	1.59	1
G-BLUP	0.27^b^ ± 0.03	0.20^b^ ± 0.03	0.70^b^ ± 0.02	1.17^b^ ± 0.13	0.40^b^ ± 0.04	0.74^b^ ± 0.22	0.77	6^b^
Pedigree	0.24 ± 0.02	0.11 ± 0.01	0.53 ± 0.02	0.87^b^ ± 0.09	0.04 ± 0.02	0.12 ± 0.06	-	1

^b^best = highest + − 0.02 for h2a, h2d, cor a, cor d and Vd/Va; 0.5 to 1.5 for bya and byd; highest minus 2 for best criteria in the last column

The results in Table 5 are for the fourth scenario and are similar to those in Table 3, with G-BLUP outperforming the three BayesA*B* methods, except in recovering the dominance/additive variation ratio. G-BLUP in particular proved to be better for estimating dominance in a mixed inheritance model scenario.

**Table 5:** Scenario 4: Results for the trait controlled by mixed (major and small gene effects) inheritance model with heritability 0.50

Method	h2a	h2d	cor_a	byg_a	cor_d	byg_d	Vd/Va	Number of criteria best
Parametric	0.33 ± 0.01	0.21 ± 0.01	0.69	-	0.55	-	0.64	-
BRR (−2,-2)	0.25^b^ ± 0.06	0.17 ± 0.04	0.69^b^ ± 0.02	1.36^b^ ± 0.24	0.42 ± 0.03	0.83^b^ ± 0.18	0.67^b^	5
IBLASSO (4,-2)	0.24^b^ ± 0.07	0.18 ± 0.04	0.69^b^ ± 0.02	1.44^b^ ± 0.30	0.41 ± 0.04	0.79^b^ ± 0.20	0.74	4
IBLASSO (4,2)	0.25^b^ ± 0.07	0.15 ± 0.04	0.69^b^ ± 0.03	1.35^b^ ± 0.27	0.42 ± 0.04	0.90^b^ ± 0.26	0.61^b^	5
BAYESA*B* (−2,6)	0.26^b^ ± 0.07	0.14 ± 0.03	0.69^b^ ± 0.03	1.31^b^ ± 0.26	0.42 ± 0.04	0.97^b^ ± 0.03	0.55	4
BAYESA*B* (4,6)	0.26^b^ ± 0.07	0.14 ± 0.04	0.69^b^ ± 0.03	1.31^b^ ± 0.26	0.42 ± 0.04	0.96^b^ ± 0.28	0.55	4
BAYESA*B* (−2,8)	0.26^b^ ± 0.07	0.14 ± 0.04	0.69^b^ ± 0.03	1.29^b^ ± 0.25	0.42 ± 0.04	0.99^b^ ± 0.30	0.53	4
RR-HET (-2,–2)	0.23 ± 0.07	0.17 ± 0.04	0.69^b^ ± 0.02	1.44^b^ ± 0.30	0.41 ± 0.04	0.80^b^ ± 0.20	0.74	3
BLASSO (4,2)	0.23 ± 0.08	0.21 ± 0.06	0.68^b^ ± 0.03	1.37^b^ ± 0.35	0.41 ± 0.03	0.86^b^ ± 0.26	0.88	4
G-BLUP	0.25^b^ ± 0.06	0.19 ± 0.04	0.70^b^ ± 0.02	1.25^b^ ± 0.03	0.46^b^ ± 0.02	0.94^b^ ± 0.20	0.76	6
Pedigree	0.20 ± 0.02	0.13 ± 0.01	0.45 ± 0.03	0.84^b^ ± 0.11	0.08 ± 0.03	0.24 ± 0.10	-	1

^b^best = highest + − 0.02 for h2a, h2d, cor a, cor d and Vd/Va; 0.5 to 1.5 for bya and byd; highest minus 2 for best criteria in the last column

### Partition of accuracy due to the three quantitative genetics information sources

The results referring to partitioning of the quantitative genetic information for h^2^ = 0.5 and a mixed inheritance model are presented in Table 6 (method BayesA*B* (−2,8)).

**Table 6:** Partition of accuracy due to the three quantitative genetics information for a trait controlled by mixed (major and small gene effects) inheritance model with heritability 0.50 (method BayesA*B* (−2,8))

Information	Additive h^2^	Composition of information	Additive accuracy	Composition of accuracy
1: Raw	0.26	COSEG+ IBD-LD + F-IBD-R	0.69	Calculated from data
2: AWF	0.22	COSEG + LD	0.53	Calculated from data
3: DMS	0.16	LD	0.52	Calculated from data
4: (2) minus (3)	0.06	COSEG	0.10	Sqr(0.53^2^–0.52^2^)
5: (1) minus (2)	0.04	F-IBD-R	-	-
6: Pedigree-Raw	0.20	COSEG + I-IBD-R	0.45	Calculated from data
7: (6) minus (4)	0.14	I-IBD-R	0.43	Sqr(0.45^2^–0.10^2^)
9: Parametric	0.33	ALL	-	-

*I-IBD-R* individual IBD relationships, *F-IBD-R* family IBD relationships, *Sqr* square root

From the genomic heritability (0.26), it can be seen that the main source of information is LD (0.16), followed by co-segregation (0.06) and family IBD relationships not linked to QTL (0.04). In the simulation, the proportion (r^2^_mq_) of genetic variation explained by markers exclusively in LD was high, approximately 90 %. In such a case, genetic variation is mainly due to LD rather than co-segregation and residual polygenic effects; thus, the results are corroborated.

From the pedigree heritability (0.20), it can be seen that the main source of information is individual IBD relationships (0.14), which was a fraction (0.875 = 0.14/0.16) of the IBS-LD captured by markers, followed by co-segregation (0.06). These partitions are in accordance with results reported by [28]. Not all of the 0.14 value necessarily originated from the 0.16, as the pedigree can capture some loci that markers cannot. Accuracy estimates follow almost the same tendency.

The additive accuracy of related individuals (*r*_*gĝr*_ using the raw dataset) was 0.69. It can also be given as a function of accuracy due to pedigree (*r*_*gĝ*ped_) and the accuracy of unrelated individuals (*r*_*gĝu*_) by the following: *r*_*gĝr*_ = *r*_*gĝped*_ + (1 ‐ r_*gĝ*ped_)*r*_*gĝu*_ = 0.45 + (1 − 0.45) 0.52 = 0.73, which is close to 0.69. It can be observed that the use of related individuals increases the accuracy.

As G-BLUP cannot capture short-range LD information well, [28] recommended Bayesian methods with t-distributed priors that are expected to capture LD better than G-BLUP [48]. Our results support those conclusions by showing that BayesA*-B*, which uses t-distributed priors, was the best for recovering the dominance variance/additive variance ratio (Tables 2, 3 and 4).

## Discussion

**Fig. 2:**
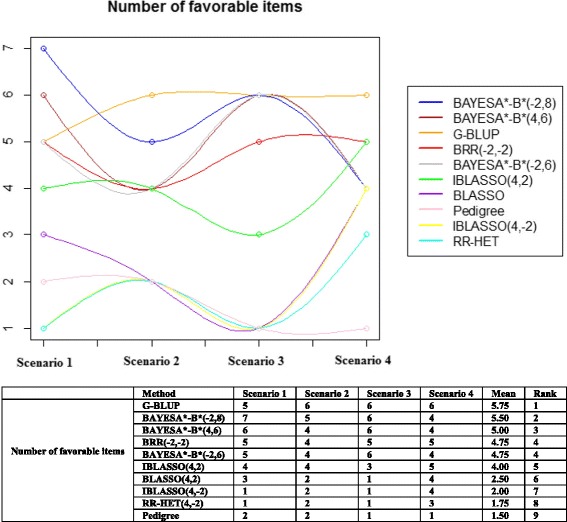
Comparison of methods in terms of the number of favorable items in the four scenarios

The so called BayesA*B* methods fitted by the GS3 software produced the best results, together with G-BLUP. The degrees of freedom associated with prior error variance were found to have little impact in the three BayesA*B* methods, and the greater impact comes from using adequate (6 or 8 instead of −2, 2 or 4) degrees of freedom for the marker variance associated with the shrinkage parameter. Using 6 or 8° of freedom produced only small differences, the BayesA*B* (−2, 8) being slightly better. G-BLUP was as good as these BayesA*B* methods. Figure 2 and the associated table summarize the results and show the following final classification of methods: (i) best: G-BLUP; BAYESA*B* (−2,8); BAYESA*B* (4,6); (ii) intermediate: BRR (−2,-2); BAYESA*B* (−2,6); IBLASSO (4,2); and (iii) worst: IBLASSO (4,-2); RR-HET (-2–2); BLASSO (4,2); Pedigree.

In general, the Bayesian Ridge Regression (BRR) method provided good results. This finding is in accordance with [40], who reported that the Bayesian Ridge model with marker-homogeneous shrinkage was among the models with the highest predictive ability in all datasets. Additionally, they found that, independent of the number of markers and observations, marker-specific shrinkage did not outperform marker-homogeneous shrinkage. Considering the higher computing efforts of models with marker-specific shrinkage, they recommended the Bayesian Ridge method as a robust model for genome-based prediction. In line with this recommendation, most studies report that Bayesian shrinkage models perform as well as or slightly better than the G-BLUP model (equivalent to the ridge regression model).

In BayesA and BayesB, the degrees of freedom of the fully conditional posterior distribution of *σ*_*mai*_^2^ are df + 1 (where df is the prior degrees of freedom). Thus, it is only one degree of freedom higher than the prior degrees of freedom, independent of the number of observations (N) or markers (n) in the model [2] and [40]. However, in the Bayesian Ridge Regression, the degrees of freedom increase with the number of markers in the model. In genomic datasets, learning in the Bayesian methods is limited due to the n> > N situation. With next generation sequencing data, n will be even larger and is expected to increase to much more than N. Thus, models with a strong Bayesian learning ability such as the Bayesian Ridge and Bayesian Lasso will be useful [40].

The accuracies were very close across the methods for all effects (additive and dominance, although dominance effects were poorly estimated). This result is in accordance with the results in the literature [38, 45], which indicate the similarity of several methods in terms of accuracy for predictive purposes. Thus, the main criteria contributing to the differences among the methods are bias (related to architecture learning), heritability estimation and dominance/additive variation capture.

The IBLASSO (4,-2) method, criticized by [38] in terms of the chi-square number (−2) of degrees of freedom for marker variance, also performed poorly in the present work, as did the RR-BLUP-HET method that used variance component results from the same IBLASSO (4,-2) method. In an attempt to improve the results, in the case of the BLASSO used by [49], the degrees of freedom of the chi-square prior distributions for genetic variances were changed from −2 to 2, producing the IBLASSO (4,2) method. This method was better than IBLASSO (4,-2) but worse than BLASSO (4,2) fitted in the BLR software.

For the estimation methods, 7 evaluation criteria were used. The accuracy did not differ much, even with contrasting methods, corroborating the majority of the reports in the literature [13, 14, 38, 45]. Unbiasedness and learning of the genetic architecture favored the methods fitted through Bayesian Lasso.

Across the 7 criteria, the additive-dominance BayesA*-B*-type or t-BLASSO methods (with 6 or 8° of freedom on a chi-square distribution for genetic variance and then for the penalization parameter) and G-BLUP performed best in over 5 criteria.

With increasing degrees of freedom in the chi-square distribution for variance components, the DE distribution for marker effects goes to a normal distribution, with the t distribution between them. Because the Student t-distribution approximates the normal distribution when the degree of freedom v increases, G-BLUP can be considered a limiting case of BayesA. The fitting of the BLASSO with new double exponential and t distributions has been considered recently [46]. They proposed three new methods (improved double-exponential prior, improved Student’s t prior and extended Bayesian LASSO) that outperformed the traditional Bayesian LASSO. The Bayes/Blasso models that we fitted differed in the prior specification for the marker effects, with hyperparameters controlling the amount of shrinkage of the effects. Because the degree of freedom v controls the thickness of the tails of a t-distribution, the choice of v had a large effect on the results.

Fang et al. [46] reported that Bayesian LASSO usually cannot effectively shrink the zero-effects QTL very close to zero. They concluded that the improved Student’s t prior for the LASSO is able to effectively shrink the zero-effects QTL toward zero, and the signals of the QTL were very clear. The results reported by [46] corroborate our choice to change the DE to a t distribution in Blasso.

In our paper, the additive-dominance BayesA*-B*-type methods that used t-distributed priors were the best for recovering the dominance variance/additive variance ratio (Tables 2, 3 and 4). This property is of great relevance for keeping the true proportionality between dominance and additive effects in the estimates. BLASSO is a better learner than BayesA and B, and it is perhaps because of this learning ability that the BayesA*B* of the present paper fitted very well, adequately recovering the parametric values.

The BRR method was the best in this criterion in one situation (Table 5). The ability to recover the heritabilities can be more sensitive to discriminate methods. This improved sensibility is because heritabilities are more complex parameters than the simple correlation coefficients (accuracies) [49]. According to [50], heritability can be regarded as a measure of the goodness of fit in the current dataset (projected to the base population), and predictive accuracy refers to prediction in future samples. Both are interdependent, and the predictive accuracy (estimated by using a validation population) is able to capture over-fitting. The heritability estimates the proportion of phenotypic variance accounted for by true genetic values in the base population comprised of unrelated individuals. On the other hand, the squared predictive accuracy estimates the proportion of phenotypic variance accounted for by predicted genetic values in the sample, not in the base population. Thus, it ignores inbreeding, relationships between individuals and estimation errors, and does not produce consistent information about the magnitude of the heritability [50].

The most probable true symmetrical distributions of genetic effects (genetic architecture) are normal (Gaussian), t (Studentian) and double exponential (Laplacean). Thus, it is imperative to test these three distributions by assuming them as priors in the methods of analyses. This approach will reveal which assumed prior distribution is more adequate and/or robust. Lehermeier et al. [40] reported that little is known about the sensitivity of the Bayesian models with respect to prior and hyperparameter specification, as comparisons of predictive performance are mainly based on a single set of hyperparameters. Our paper has varied these hyperparameters and showed that measurable differences are the result of different specifications. This finding is in accordance with the literature. BayesA and BayesB hyperparameter settings had a stronger effect on predictive performance than was observed with the Blasso and Bayesian regression [40].

Wang et al. [24] presented the traditional quantitative genetics model as the unifying model for definitions of the genomic relationship and inbreeding coefficients. Under the correct definitions of these coefficients, the G-BLUP procedure seems to suffice. According to them, theoretical differences between the existing and new definitions of genomic additive and dominance relationships were in the assumptions of equal SNP effects (equivalent to across-SNP standardization), equal SNP variances (equivalent to within-SNP standardization), and expected or sample SNP additive and dominance variances. These conclusions came to facilitate the understanding and comparison of alternative prediction and estimation methods.

As advocated by [24], after their results, the need for methods comparisons is less evident. Our results showing the equivalence between several predictive methods corroborate their findings.

## Conclusions

Amongst the 10 models/methods evaluated, the G-BLUP, BAYESA*B* (−2,8) and BAYESA*B* (4,6) methods exhibited the best results and were found to be adequate for accurately predicting genomic breeding and total genotypic values, as well as for estimating additive and dominance in additive-dominance genomic models.

## Abbreviations

GWS: Genome wide selection
QTL: Quantitative trait loci
LD: Linkage disequilibrium
CS: Co-segregation
PR: Pedigree relationships
GWAS: Genome wide association studies
